# Supplementary material for: Reversed Mirror Therapy (REMIT) after Stroke—A Proof-of-Concept Study
Source: Brain Sci. 2023 May 24;13(6):847. doi: 10.3390/brainsci13060847 (PMC10296617; doi:10.3390/brainsci13060847)
Supplement: Supplementary file 1 [file brainsci-13-00847-s001.zip › Brain Sci suppl mat 01.pdf]

Supplemental Materials S1

Videos of the 19 exercises practised in conventional mirror therapy (MIT) and reversed mirror therapy (REMIT; see .mp4 files). A healthy subject completes the exercises.

The 19 exercises consisted of a-finalistic movements following the Fugl-Meyer assessment principles. Only the exercises performed with the right upper limb are shown.

List of exercises:

- Exercise 1: shoulder flexion and extension
- Exercise 2: shoulder abduction and adduction
- Exercise 3: elbow flexion and extension
- Exercise 4: forearm pronation and supination
- Exercise 5: forearm displacement on the table
- Exercise 6: shoulder internal and external rotation
- Exercise 7: hand from table to ear
- Exercise 8: forearm pronation and supination on the table
- Exercise 9: wrist abduction and adduction
- Exercise 10: wrist flexion and extension
- Exercise 11: thumb abduction and adduction
- Exercise 12: wrist flexion and extension with the prone hand
- Exercise 13: fingers flexion and extension
- Exercise 14: three knocks on the table
- Exercise 15: precision grip
- Exercise 16: finger purse supinated
- Exercise 17: finger purse on the side
- Exercise 18: 2nd finger extension and flexion
- Exercise 19: single fingers extension and flexion

Two representative videos of a patient exercising with the reversed mirror therapy (REMIT) are also provided.

The patient, affected by left hemiparesis, is asked to exercise with both upper limbs while looking at the left (paretic) hand reflected in the mirror.

Exercises 12 (extension and flexion movements of the wrist with the prone hand) and 13 (fingers flexion and extension) are shown.

Each exercise is repeated for one minute, with a 15 – 30 seconds pause between repetitions.
