# Supplementary material for: Reversed Mirror Therapy (REMIT) after Stroke—A Proof-of-Concept Study
Source: Brain Sci. 2023 May 24;13(6):847. doi: 10.3390/brainsci13060847 (PMC10296617; doi:10.3390/brainsci13060847)
Supplement: Supplementary file 1 [file brainsci-13-00847-s001.zip › Brain Sci suppl mat 02.pdf]

Supplemental Materials S2

Association between motor cortex excitability and post-treatment improvements

The PCA was used to evaluate the association between the changes in the upper limb dexterity measures and the measures of corticospinal excitability obtained with the transcranial magnetic stimulation (TMS) of the hand motor cortex at baseline.

The following measures were included in the dataset for the PCA:

1. Difference between the upper-limb Fugl-Meyer measure (ul-FM) at the end of the first week of treatment (T1) and baseline (T0; ul-FM $\Delta_{T1\text{-}T0}$),
2. Difference between ul-FM at the end of the second week of treatment (T2) and baseline (ul-FM $\Delta_{T2\text{-}T0}$),
3. Resting motor threshold (RMT) of the motor evoked potentials (MEP) of the First Dosal Intersosseus (FDI) muscle,
4. Peak-to-peak amplitude of the FDI MEP elicited at 1.1 times the RMT and
5. Area of the FDI cortical representation.

The three neurophysiological parameters were measured in the good hand and the paretic.

Table SM2-1.

	**eigenvalue**	**% of variance**	**cumulative % of the variance**
**PC1**	3.59	44.8	44.8
**PC2**	2.46	30.8	75.6
PC3	0.86	10.7	86.4
PC4	0.79	9.9	96.3
PC5	0.19	2.3	98.6
PC6	0.11	1.4	100.0

Two principal components (PC) had eigenvalue >1 (PC1 and PC2, bold). Altogether, PC1 and PC2 explained about 75% of the total variance of the dataset.

Table SM2-2.

PC1

	**correlation**	**P-value**	**P-value, Holm**
**Area, sound**	0.792	0.019	0.038
RMT, impaired	-0.625	0.098	0.098
**ul-FM** Δ_{T1-T0}	-0.872	0.005	0.014
**ul-FM** Δ_{T2-T0}	-0.944	0.000	0.002

PC2

	**correlation**	**P-value**	**P-value, Holm**
RMT, impaired	0.666	0.071	0.089
MEP, impaired	-0.719	0.044	0.089
**Area, impaired**	-0.961	0.000	0.000

The variables strongly correlated with PC1 and PC2 (i.e., those with a correlation coefficient outside the -0.6 to 0.6 range) are reported. The Type I error probability of these correlations is also given. P-value, Holm: Type I error probability corrected for multiplicity according to Holm. Sound: unimpaired hand; impaired: paretic hand.

PCA, correlations between variables and PCs and their P-values were calculated with the FactoMineR library[1] in R version 3.6.2.

References

1. Lê S, Josse J, Husson F. FactoMineR: A Package for Multivariate Analysis. *Journal of Statistical Software*. 2008;25:1–18.
